# Chemical Composition, Antioxidant, Antimicrobial and Cytotoxic/Cytoprotective Activity of Non-Polar Extracts of Grape (*Vitis labrusca* cv. Bordeaux) and Blackberry (*Rubus fruticosus*) Seeds

**DOI:** 10.3390/molecules26134057

**Published:** 2021-07-02

**Authors:** Tufy Kabbas Junior, Cristiane de Moura, Mariana Araújo Vieira do Carmo, Luciana Azevedo, Luis Antônio Esmerino, Rosangela Capuano Tardivo, Petri Kilpeläinen, Daniel Granato

**Affiliations:** 1 Graduate Program in Chemistry, State University of Ponta Grossa (UEPG), Av. Carlos Cavalcanti, 4748, Ponta Grossa 84030-900, Parana, Brazil; tufy_kj@hotmail.com (T.K.J.); tiane.moura@hotmail.com (C.d.M.); 2 Graduate Program in Biosciences Applied to Health, Federal University of Alfenas (UNIFAL), R. Nabor Toledo Lopes, 598-Parque das Nações, Alfenas 37130-000, Minas Gerais, Brazil; marianavieira06@hotmail.com (M.A.V.d.C.); lucianaazevedo2010@gmail.com (L.A.); 3 Department of Clinical Analysis, State University of Ponta Grossa, Av. Carlos Cavalcanti, 4748, Ponta Grossa 84030-900, Brazil; esmerino@uepg.br; 4 Department of Biology, State University of Ponta Grossa, Av. Carlos Cavalcanti, 4748, Ponta Grossa 84030-900, Brazil; rc.tardivo@uol.com.br; 5 Production Systems & Biorefinery & Bioproducts, Natural Resource Institute, Finland (Luke), Tietotie 2, FI-02150 Espoo, Finland; petri.kilpelainen@luke.fi; 6 Department of Biological Sciences, Faculty of Science and Engineering, University of Limerick, V94 T9PX Limerick, Ireland

**Keywords:** cell models, antioxidants, fruit seeds, fatty acids, in vitro bioactivity

## Abstract

The aim of this study was to compare the influence of the extraction method, chemical composition, antimicrobial effects, antioxidant activity, and cytotoxicity on human cells of the non-polar extracts of grape (*Vitis labrusca*) and blackberry (*Rubus fruticosus*) seeds. The Soxhlet (Sox), Bligh–Dyer (BD), and ultrasound (US) methods were used for extractions. For blackberry non-polar seed extract, extraction via the BD method showed the highest mean values of total phenolic content (TPC), expressed in milligrams of gallic acid equivalent per 100 mL of non-polar seed extracts (102.37 mg GAE/100 mL), and higher antioxidant activity in relation to the 2,2-diphenyl-1-picrylhydrazyl (DPPH) radical, expressed in milligrams of gallic acid equivalent per 100 mL of non-polar seed extracts (11.50 mg AAE/100 mL), if compared with the Sox and US extractions. Similar results were obtained for the non-polar grape seed extracts, where BD extraction obtained the highest values for TPC (28.61 mg GAE/100 mL) and DPPH (35.36 mg AAE/100 mL). The type of extraction method had an impact on the composition of fatty acids. Only the non-polar blackberry and grape seed extracts obtained via the Sox method showed some in vitro inhibitory effect against *Escherichia coli* (IAL 2064) and *Staphylococcus aureus* (ATCC 13565). Regardless of the extraction method used, the non-polar blackberry and grape seed extracts did not decrease the cell viability (IC_50_ >1000 µg/mL) of cancer and normal cell lines, thus indicating the relative safety of the extracts. All the seed extracts decreased the generation of reactive oxygen species in the cell lines. Blackberry and grape seed lipid fractions can be utilized as antioxidants, and the extraction methods used cause significant changes in relation to their bioactivity and chemical composition.

## 1. Introduction

Due to their technological versatility, vegetable oils are used in the food, pharmaceutical, cosmetic, agronomic, sanitary, and fuel industries. Such oils can be extracted from flowers, leaves, branches, trunks, fruits, bark, seeds, and roots. The extraction of vegetable oils represents an important economic activity both in Brazil and worldwide, the most representative being oils extracted from soybeans (*Glycine max* L. Merrill), palm (*Elaeis guineensis* Jacq.), rice (*Oryza sativa* L.), cotton (*Gossypium hirsutum*), and sunflower seeds (*Helianthus annuus*) [1]. Species such as andiroba (Carapa guianensis), buriti (*Mauritia flexuosa*), copaiba (*Copaifera* sp.), pequi (*Caryocar brasiliense* Camb.), golden linseed (*Linum usitatissimum*), avocado (*Persea americana* cv. Butter), pomegranate (*Punica granatum*), and grapes (*Vitis labrusca* cv. Bordeaux) are also used in the production of oils in Brazil, however, these are less expressive, that is, on a smaller scale [2].

Vegetable oils are increasingly used because they present a complex mixture of compounds [3]. For example, some classes of compounds, such as fatty acids, phenolic compounds, carotenoids, tocopherols, and phytosterols, are present in vegetable oils and are responsible for antioxidant properties and beneficial health effects [2,4,5]. Triacylglycerols are formed from the combination of a glycerol molecule with three fatty acids, together with diacylglycerols, free fatty acids, and phospholipids they are the major components of edible vegetable oils (95–98%) [6]. In addition, some vegetables oils are sources of essential fatty acids such as oleic, linoleic, and linolenic acid (olive, grape, canola, sunflower, corn, soy, etc.). This chemical composition can be influenced by factors such as harvest period, climatic conditions, region of cultivation, and extraction method, all of which can affect the functional properties of oils [7].

Studies have shown that the consumption of phenolic compounds is strongly related to the reduction of the risk of cancer, inflammatory disorders and cardiovascular diseases [8]. Santos et al. [9] studied the methanol/water (90/10 *v*/*v*) extract of grape seed oil (Vitis labrusca cv. Bordeaux) obtained by cold pressing. They demonstrated antioxidant activity against the 2,2-diphenyl-1-picrylhydrazyl (DPPH) radical and 2,2′-azino-bis (3-ethylbenzothiazoline-6-sulfonic acid) (ABTS). Deolindo et al. [10] showed that hydroethanolic extracts from grape seeds (Vitis labrusca cv. Bordeaux) have chemical antioxidant activity based on the DPPH assay, as well as the in vitro potential to inhibit the activity of the angiotensin I-converting enzyme (ACE-I). Harbeoui et al. [11] confirmed the anti-inflammatory capacity of the ethanol/water extract (80/20, *v*/*v*) of Vitis vinifera grape seeds using RAW 264.7 cells. These authors also suggested that the different mechanisms involved in anti-inflammatory activity could be due to the synergism between various phenolic compounds as well as the specific structural elements of flavonoids (dominant fraction). Studies of the ethanol/acidic water extract (pH 3.2) 7:3 *v*/*v* from cold-pressed fresh grape seed oils, performed by Cecchi et al. [12], demonstrated in vitro inhibitory activity against PTP-1B (protein-tyrosine phosphatase 1B enzyme), an enzyme that is over-expressed in type 2 diabetes, with maximum inhibition values (98%) for *Vitis vinifera* cv. Sauvignon Blanc and minimum inhibition values (40%) for Vitis vinifera cv. Cabernet Sauvignon. These authors suggested that inhibitory activity was exerted by the phenolic fraction isolated from these oils, and that further studies were required to confirm the action of grape seed oils and help to understand the mechanism behind this activity.

Micić et al. [13] analyzed the hexane extract from blackberry and raspberry seeds; they identified essential fatty acids (oleic, linoleic, and linolenic acids) as the major compounds. Blackberry is a fruit of interest because of its high content of anthocyanins and ellagitannins (ETs) in the pulp, as well as other phenolic compounds that contribute to its high antioxidant capacity. Blackberry pulp has previously shown protective activity against oxidative stress agents, endotoxicity, age-related neurodegenerative diseases, obesity, cancer, diabetes, and cardiovascular diseases [14,15,16].

In order to contribute to the existing knowledge about the relationship between chemical composition and biological activities of extracts obtained from agroindustry tailings, this study aimed to examine the effect of the extraction method (Soxhlet, Bligh–Dyer, and ultrasound) on the antioxidant, antimicrobial, and cytotoxic properties on normal and cancerous cells, as well as the chemical composition of non-polar grape seed extracts (*Vitis labrusca*) and blackberry (*Rubus fruticosus*).

## 2. Results and Discussion

### 2.1. Total Phenolic Content and Antioxidant Activity

The data regarding TPC, antioxidant activity, and yield (%) are shown in Table 1. The yield obtained from the extractions were as follows: Sox (31.9 and 20.7%); BD (20.0 and 13.3%); and US (12.6 and 7.7%) for grape and blackberry seeds, respectively, in each technique used. For the non-polar extracts of blackberry seeds, extraction via BD showed the highest mean values for TPC (102.37 mg GAE/100 mL) and antioxidant activity in relation to DPPH (11.50 mg AAE/100 mL). Regarding the total reducing capacity (TRC), expressed in milligrams of quercetin equivalent (QE) per 100 mL of non-polar seed extracts, of the blackberry seed extracts, the extraction using BD (46.42 mg QE/100 mL) also showed the highest mean values (*p* < 0.05) compared to the other extractions (not detected). For the ability to chelate Fe^2+^, expressed in milligrams of ethylenediamine tetraacetic acid equivalent (EDTAE) per 100 mL of non-polar seed extracts, the highest mean values were found using extraction via Sox and US, (261.12 mg EDTAE/100 mL) and (77.57 mg EDTAE/100 mL) respectively, for blackberry extract. For the grape extract, the BD extraction showed the highest TPC (28.61 mg GAE/100 mL) and DPPH (35.36 mg AAE/100 mL). The values showed herein are lower than those found by Deolindo et al. [10], studying a Bordeaux grape seed extract obtained with ethanol/water (TPC = 3,886 ± 124 mg GAE/100 g, DPPH = 12,872 ± 64 mg AAE/100 g). The ability to chelate Fe^2+^ (grape extract) showed higher values with Sox extraction (155.01 mg EDTAE/100 mL) and US (74.70 mg EDTAE/100 mL), differing only in relation to TRC activity, which presented the greatest activity with extraction via US.

### 2.2. Composition of Fatty Acids and Phytosterols

The individual fatty acid and phytosterols contents of blackberry and grape non-polar seed extracts is shown in Table 2. There were significant differences (*p* < 0.05) between the three extraction methods: except for palmitic acid (*p* = 0.8746), linolenic acid (*p* = 0.7702), linoleic acid (*p* = 0.7155), oleic acid (*p* = 0.2824), stearic acid (*p* = 0.0605), and arachidic acid (*p* = 0.0524) for the blackberry non-polar seed extracts and linoleic ethyl ester (*p* = 0.0872) and monopalmitin (*p* = 0.1725) for the grape non-polar seed extracts. Blackberry extracted with US had the highest levels (*p* < 0.001) of β-sitosterol, β-amyrenol, and methylene cycloartenol, whereas for grape non-polar seed extracts the highest contents were arachidic acid, 9-octadecanoic acid, campesterol, stigmasterol, and β-sitosterol.

The proportion of linoleic acid (18:2) 42–52% in grape seed extract fatty acids was lower than those reported in the literature for grape (*Vitis* sp.) seed oil: 63–75% [17] and 66–75% [18]. The oleic acid (18:1) proportion was 29–37%, palmitic acid (16:0) 10–14%, stearic acid (C18:0) 3–5%, linolenic acid 2–4% (C18:3), and arachidic acid 1–3% of fatty acids in extracts.

Fatty acid proportions of blackcurrant extracts were 44–53% linoleic acid (18:2), 31–42% oleic acid (18:1), 6–9% palmitic acid (16:0), 2–5% stearic acid, 1% linolenic acid (18:3), and 1–3% arachidic acid (20:0). Proportions of linoleic acid are lower and oleic acid higher than in the literature [13], where values were 66% and 13%. Extractions were done with hexane with a different extraction set-up and at a lower extraction temperature (4 °C) than this study, which could explain the differences in proportions of fatty acids in extracts.

### 2.3. Antimicrobial Activity

Antimicrobial activity was tested against *Escherichia coli* (IAL 2064) and *Staphylococcus aureus* (ATCC 13565). The results showed that only the non-polar blackberry and grape seed extracts obtained via Sox presented some inhibitory effect. The blackberry extract inhibition values in relation to *E. coli* (IAL 2064) were 99.4%, 62.5%, 33.8%, and 33.4% for concentrations of 33.3, 1.67, 0.83, and 0.42 µg/L, respectively. For *S. aureus* (ATCC 13565), the inhibition values of the blackberry extract were 90.7%, and 33.3% for concentrations of 33.3 and 1.67 µg/L, respectively. Studies of the antimicrobial capacity of non-polar blackberry seed extracts are not reported in the literature. Weli et al. [19] studied the antibacterial activity of blackberry leaf extracts (methanol, hexane, chloroform, ethyl acetate, and hydro-alcoholic) and concluded that they are effective against several Gram-positive and Gram-negative micro-organisms.

The non-polar grape seed extract obtained via Sox showed inhibition against *E. coli* (IAL 2064) at concentrations of 33.3, 1.67, and 0.83 µg/L with values of 92.7%, 49.9%, and 34.4% inhibition and against *S. aureus* (ATCC 13565) with values of 85.1% and 36.6% inhibition at concentrations of 33.3 and 1.67 µg/L, respectively.

A study conducted by Duran et al. [20] demonstrated the antimicrobial effect of grape seeds by using chitosan-coated extracts to prolong the lifespan of fresh strawberries. The antimicrobial activity of grape pomace was described by Leal et al. [21], and the authors concluded that grape stems demonstrated antimicrobial activity, with high efficiency against Gram-positive bacteria, especially *S. aureus* and *E. faecalis*.

### 2.4. Determination of Cellular Toxicity of the Non-Polar Seed Extracts and Measurement of ROS (Reactive Oxygen Species)

**Figure 1:**
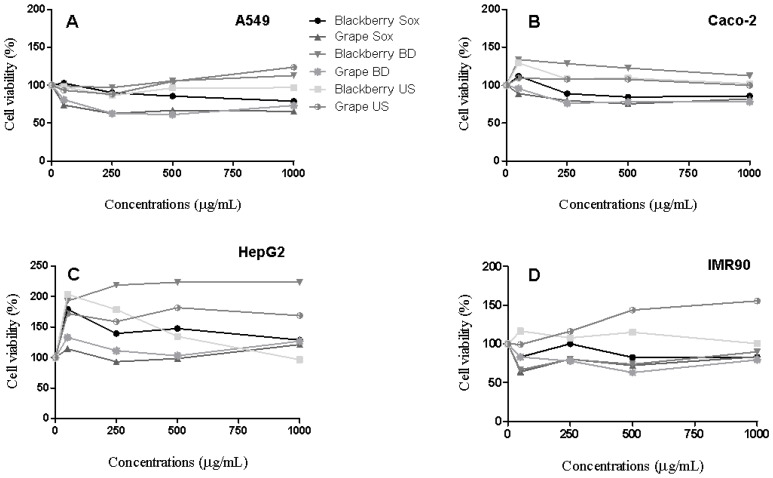
Representative cell viability of A549 (**A**) Caco-2 (**B**), HepG2 (**C**), and IMR90 (**D**) cells after 48 h exposure to non-polar blackberry and grape seed extracts.

A range of non-polar seed extract concentrations (from 100 to 1000 µg/mL) was used to assess the cell viability in A549, Caco-2, HepG2 and IMR90 cell lines. Both blackberry and grape non-polar seed extracts, independent of extraction method, did not decrease the cell viability (IC_50_ > 1000 µg/mL) for cancer and normal cell lines (Figure 1). Conversely, Montserrat-de la Paz et al. [22] exhibited that the isolated β-sitosterol, one of the main fatty acids present in the blackberry and grape non-polar seed extracts (from 1.22 to 3.66 mg/g), reduced the cell viability (IC_50_ = 79.0 µM) in HT-29 cancer cells. Taking this into account, it is important to consider that there are differences between working with individual compounds (β-sitosterol) and whole natural matrices (i.e., non-polar seed extracts). Thus, the final biological potential is not always the sum of each one of the individual compounds present in the non-polar seed extracts, as different interactions may occur in biological media [23].

**Figure 2:**
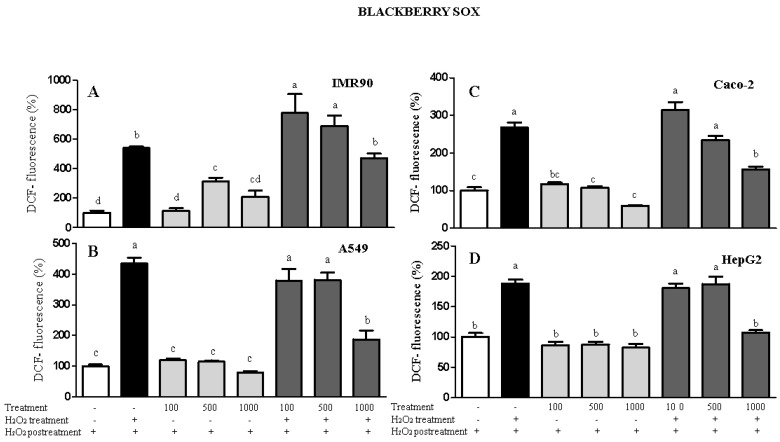

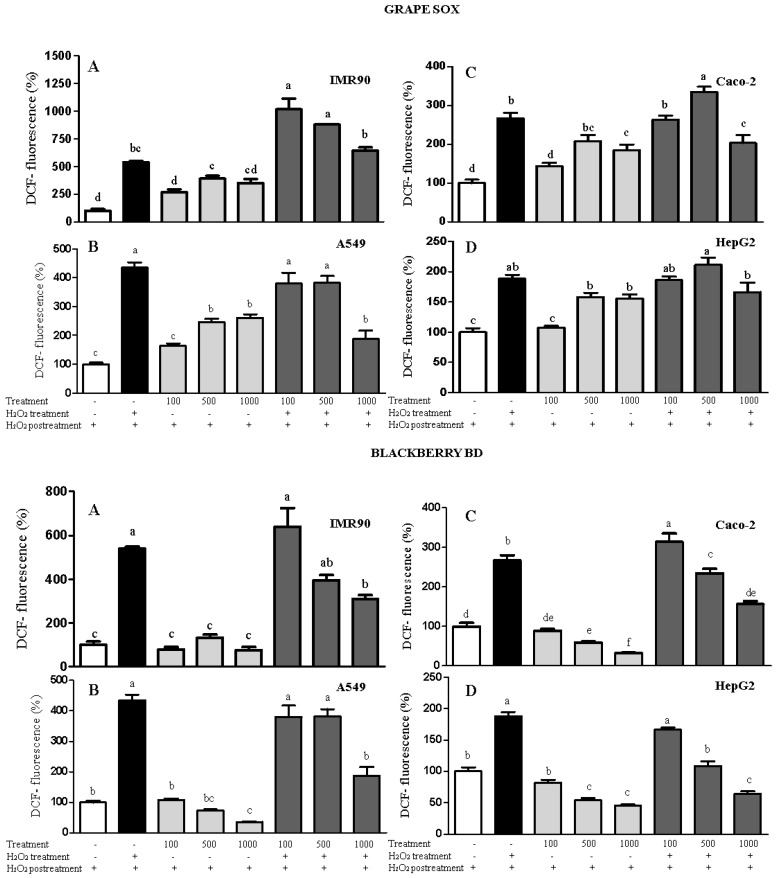

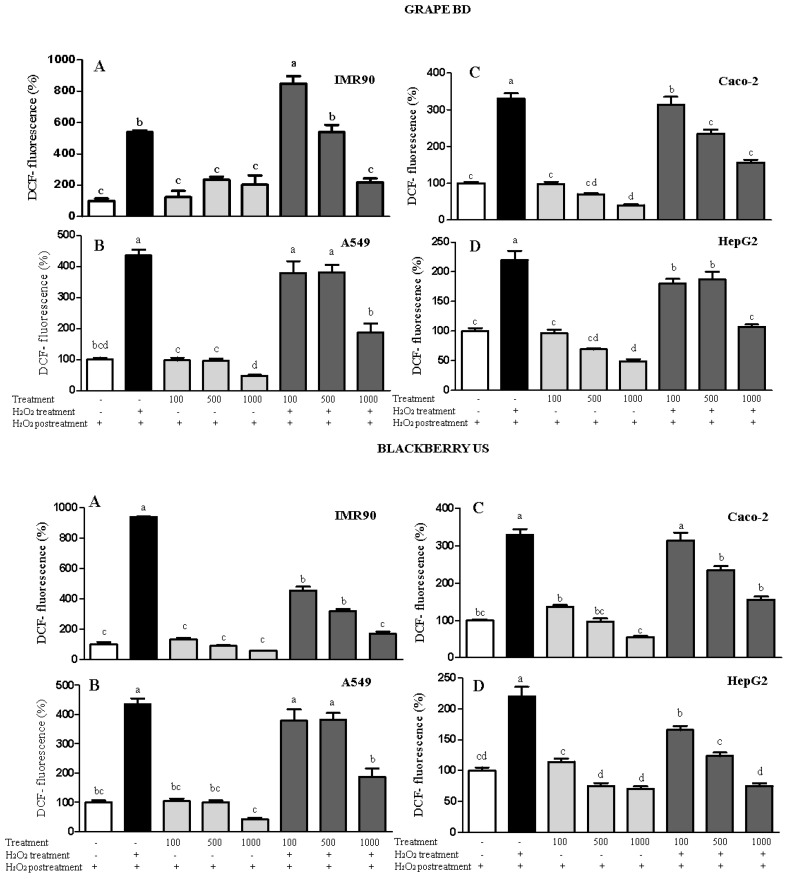

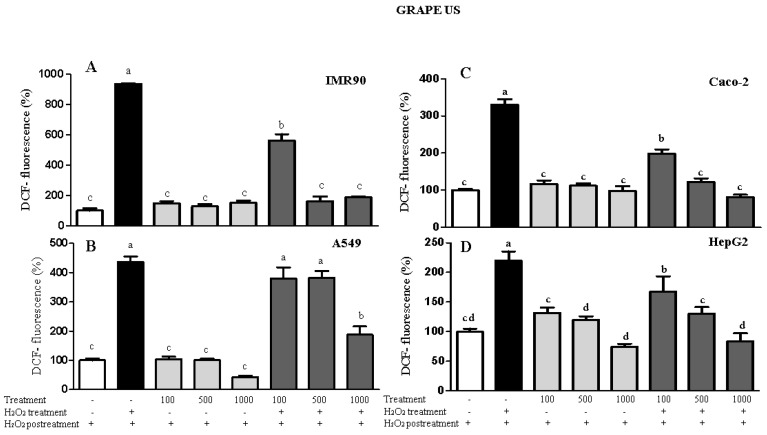
Results of the measurement of intracellular ROS in IMR90 (**A**), A549 (**B**), Caco-2 (**C**), and HepG2 (**D**) cells via spectrofluorimetry. Treatment = non-polar blackberry and grape seed extracts at 100–1000 μg/mL. Different letters comparing the treatments represent statistically different results at *p* < 0.05.

Regarding the intracellular antioxidant capacity (Figure 2), the US and Sox blackberry and grape non-polar seed extracts obtained via US were not able to induce ROS generation in all cell lines. When H_2_O_2_ was added, the non-polar seed extracts decreased the intracellular ROS levels and, in some cases, the ROS generation was similar to that of the negative control group. In contrast, grape non-polar seed extracts obtained via Sox promoted pro-oxidant behavior by inducing ROS production in both cancer and normal cells. This finding may be associated with the antioxidant activity measured using chemical methods, especially the TRC assay. Interestingly, the TRC levels were not detected for both blackberry and grape extracts obtained via Sox. In contrast, grape and blackberry non-polar seed extracts obtained using the BD method exhibited high antioxidant capacity by decreasing the ROS rate by up to 32% or maintaining their levels similar to the control. Similarly, Atolani et al. [24] showed that Citrus sinensis non-polar seed extracts also reduced the ROS rate by 50% when compared with the negative control (no addition of the oxidant agent). Additionally, Kouka et al. [25] pointed out that the phenolic fraction of olive oil did not affect the ROS levels in C2C12 and HeLLa cell lines, apart from HepG2, where ROS decreased significantly. Thus, in the present study, the intracellular antioxidant potential of grape and blackberry seed extracts obtained via BD extraction was increased for IMR90, A549, HepG2, and Caco-2 cells. This result is in agreement with the higher DPPH scavenging activity (11.50 ± 0.44 and 35.3 ± 0.99 mg AAE/100 mL for blackberry and grape seed extracts, respectively) and TPC content (102.37 ± 4.38 and 28.61 ± 0.5 mg GAE/100 mL for blackberry and grape seed extracts, respectively) displayed herein for the BD method.

## 3. Materials and Methods

### 3.1. Reagents

Folin–Ciocalteu reagent, isobutanol, quercetin (95% purity), sodium hydroxide (NaOH), 2,2-diphenyl-1-picryl-hydrazyl radical (DPPH), pyrocatechol violet (3,3′,4-trihydroxyfuchsone-2′-sulfonic acid), 2,4,6-tris (2-pyridyl)-S-triazine (TPTZ), hydrogen peroxide, hyeneicosanoic acid (C:21), cholesterol, dimethyl sulfoxide (DMSO), 2′,7′-dichlorofluorescin diacetate (DCFH-DA), ferric chloride hexahydrate, ascorbic acid, and ferrozine 3-(2-Pyridyl)-5,6-di (2-furyl)-1,2,4-triazine-5′,5′′-disulfonic acid disodium salt) were obtained from Sigma-Aldrich (São Paulo, Brazil). Anhydrous sodium sulfate, n-hexane, chloroform, and methanol were obtained from Anhydrol (São Paulo, Brazil). Ethanol was purchased from Neon (São Paulo, Brazil). The A549, IMR90, HepG2 and Caco-2 cells were obtained from the Cell Bank of Rio de Janeiro, Brazil.

### 3.2. Plant Materials

Blackberry (*Rubus fruticosus*) and purple grape (*Vitis labrusca* cv. Bordeaux) were grown in Vacaria, Rio Grande do Sul (29°32′30″ S and 50°54′51″ W) and Garibaldi, Rio Grande do Sul (29°15′22″ S and 51°32′01″ W), Brazil. The fruits (blackberry and grape), together with the leaves and flowers, were identified morphologically and exsiccates of plant material were deposited in the Herbarium of the State University of Ponta Grossa, Paraná, Brazil, under numbers 22,494 and 22,495, respectively. The grape seeds were obtained directly from a grape juice producer and the material was dried (35 °C/48 h) and ground (Tyler 60 mesh sieve). The blackberry seeds were obtained manually and went through the processes of sanitizing using NaOCl at 100 mg/L for 15 min, washing (running water), drying (greenhouse with air circulation (Tecnical, Model TE-393/1, São Paulo, Brazil) at 35 °C for 48 h, grinding (analytical mill (QUIMIS-6298A21), standardization (Tyler 60 mesh sieve), and storage under refrigeration.

### 3.3. Extraction Procedure

In order to compare the chemical composition and bioactivity of the extracts, three methodologies were tested to obtain the non-polar extracts: Soxhlet, Bligh–Dyer and ultrasound.

#### 3.3.1. Extraction with Hot Solvent (Soxhlet)

Five grams of the ground sample were weighed and transferred to the extraction cartridge. The extractions were performed in periods of 4–5 h in a Soxhlet apparatus with n-hexane as the extraction solvent. The temperature was kept constant in the extraction apparatus within the boiling range of n-hexane (68–70 °C). The extract was evaporated to remove the solvent through a vacuum evaporator (35 °C).

#### 3.3.2. Extraction Using the Bligh–Dyer Method

For the extraction, approximately 5.0 g of each sample were weighed. In an Erlenmeyer flask, 20 mL of methanol, 10 mL of chloroform, and 8 mL of water (volume dependent on the moisture content of the sample) were added. After 30 min of stirring, an additional 10 mL of chloroform and 10 mL of 1.0% *w*/*v* sodium sulfate solution were added. The mixture was stirred again for 2 min. The lower layer was removed and approximately 1.0 g of anhydrous sodium sulfate was added to remove traces of water, followed by filtration with qualitative filter paper. The solvent was removed using a vacuum evaporator.

#### 3.3.3. Ultrasound-Assisted Extraction

Ultrasound equipment (Unique, model USC-1450A, Brazil), at 130 W and 20 kHz, was used for ultrasound-assisted extraction in pulse mode. A 5.0 g sample was weighed and mixed with 100 mL of hexane. During the extraction process the container was kept in a thermostat-controlled water bath (25 °C). In all the experiments, the extracts were collected after 40 min. The resulting extracts were evaporated using a vacuum evaporator.

### 3.4. Total Phenolic Content and Antioxidant Activity

After obtaining the non-polar seed extracts, a liquid–liquid extraction was performed following the procedures described by de Santana et al. [26], with modifications. For 250 μL of non-polar seed extracts, an aliquot of 500 μL of methanol and water, in the proportion of 90/10 *v*/*v*, was added to polystyrene microtubes, and the content was vortexed for 5 min. The upper phase was collected and the non-polar seed extracts were extracted again. The extracts were kept at −18 °C.

The total phenolic content was evaluated using the Folin–Ciocalteu assay following the methodology described by Margraf et al. [27]. TPC was expressed in milligrams of gallic acid equivalent per 100 mL of non-polar seed extracts (mg GAE/100 mL). The capture of the DPPH radical was evaluated in a buffered system at pH 6, using the experimental conditions described by Santos, Brizola and Granato [28]. The absorbance was at 525 nm and the data were expressed in milligrams of ascorbic acid equivalent per 100 mL of non-polar seed extracts (mg AAE/100 mL).

The determination of the TRC of the extracts was based on the Folin–Ciocalteu method modified by Berker et al. [29]. The absorbance was at 665 nm, using a microplate reader (Biotek, model Epoch, Winooski, VT, USA). The analyses were performed in triplicate and the results were expressed in milligrams of quercetin equivalent per 100 mL of non-polar seed extracts (mg QE/100 mL).

The Fe^2+^ chelating ability of the extracts was evaluated using the colorimetric method proposed by Carter [30], adapted to the microplate reader [28]. The formation of the complex was calculated by Equation (1). The results were expressed in milligrams of ethylenediamine tetraacetic acid equivalent per 100 mL of non-polar seed extracts (mg EDTAE/100 mL).

Fe^2+^ chelating capacity (%) = ((Abs sample − Abs blank)/Abs control) × 100 (1)

### 3.5. Chemical Composition by Gas Chromatography–Mass Spectrometry (GC-MS)

For the GC-MS analysis, each sample (1 mL) was silylated by adding 100 μL pyridine, 100 μL of N,O-Bis(trimethylsilyl)trifluoroacetamide (BSTFA), and 50 μL of trimethylsilyl chloride (TMCS). The samples were analyzed using an HP 6890 GC system equipped with a 7683 automatic sampler, a 5973 selective mass detector, and a Zebron ZB SemiVolatiles capillary column (30 m × 0.25 mm id; 0.25 μm film thickness). Helium was used as carrier gas at a flow rate of 1.5 mL/min. Heneicosanoic acid (C:21) and cholesterol were used as internal standards. The GC-MS used the following conditions: initial temperature of 150 °C, temperature rate 7 °C/min for temperature 230 °C and 4 °C/min for temperature 290 °C, waiting time 15 min, injector temperature 280 °C and 1:10 ratio, MS interface temperature of 300 °C, ion source temperature of 230 °C, and quadrupole temperature of 150 °C. Results were calculated with the internal standard and were in the linear range of the MS detector. Repeatability of the method, expressed by the relative standard deviation between analyses, was below 5%. The mass spectra were obtained via ionization energy by electronic impact (EI mode) 70 eV between *m*/*z* 50 to 800, following the method described by Väisänen et al. [31]. The fatty acids and phytosterols were identified by using the NIST14 and Wiley11 mass spectrometry libraries, as well as the laboratory’s own library. The identification results were compared with the known retention times of the analytes and were expressed in milligrams per milliliter of the non-polar seed extract (mg/mL).

### 3.6. Antimicrobial Activity

#### 3.6.1. Minimum Inhibitory Concentration (MIC)

The minimum inhibitory concentration (MIC) of the extracts was evaluated according to the microdilution plate technique, using 96-well microplates, adapted from the Clinical Laboratory Standards Institute (CLSI, 2014). The extracts were tested in concentrations of 3.33, 1.67, 0.83, and 0.42 µg/L against *E. coli* (IAL 2064) and *S. aureus* (ATCC 13565). The tests were standardized in sterile physiological solution equivalent to a 0.5 McFarland standard. The negative controls were the culture medium, and the positive controls were the culture medium added to the tested microorganism. The plates were incubated at 35 °C for 24 h. The antimicrobial activity was calculated by the differences between the average of the optical density readings (OD) of the sample and its blank spaces (three readings were recorded after shaking at 620 nm). Inhibition values were considered significant above 30% and *p* < 0.05.

#### 3.6.2. Cytotoxicity Analysis

##### Cell Viability

The in vitro cell viability of grape and blackberry non-polar seed extracts was evaluated in relation to A549, IMR90, HepG2, and Caco-2 cell lines via the MTT assay [32]. Briefly, cells (1 × 10^4^ cells/well) were seeded into 96-well microplates and treated for 48 h with 50, 250, 500, and 1000 μg/mL of grape and blackberry non-polar seed extracts, which were diluted in DMSO and Tween 80 at final concentrations of 0.35% and 0.0009%, respectively, for all wells. The optical density (OD) was measured at 570 nm and percent cell viability relative to the control was calculated using the following formula, (%) = (OD_treatment_ − OD_blank_)/(OD_control_ − OD_blank_) × 100. The data were used to calculate IC_50_ values by non-linear regression.

##### Cellular Antioxidant Activity

The intracellular antioxidant potential of grape and blackberry nonpolar seed extracts was evaluated in A549, Caco-2, HepG2 (6 × 10^4^ per well) and IMR90 (2 × 10^4^ per well) cell lines. Cells were treated with 100, 500, and 1000 μg/mL of extracts diluted in a 25 mmol/L DCFH-DA (2′,7′-dichlorofluorescin diacetate) solution with and without 15 μmol/L H_2_O_2_. Cells were then incubated at 37 °C for 1 h with the treatments and subsequently they were washed with PBS and an H_2_O_2_ solution (15 μmol/L) was added in the wells. The positive control was treated with 15 μmol/L H_2_O_2_ and negative control only with culture medium. The fluorescence intensity was measured at an excitation wavelength of 485 nm and at an emission wavelength of 538 nm. The data were expressed as percentage of fluorescence intensity [33].

### 3.7. Statistical Analysis

The experimental data were presented as rates ± sample standard deviation. When appropriate, the comparison of rates between the groups was performed by a unifactorial analysis of variance (ANOVA), followed by a Fisher’s multiple comparison test. For this, the homoscedasticity of the entire data set was formally verified using the Brown–Forsythe test using the TIBCO Statistica 13.3 (TIBCO Software Ltd., Palo Alto, CA, USA) software.

To correlate the bioactivity with the chemical composition of the non-polar seed extracts, the correlation coefficients were calculated. This analysis helps to understand which phenolic compound is responsible for the functional property in vitro [34].

## 4. Conclusions

The non-polar seed extracts obtained using the BD method showed the highest levels of total phenolic compounds and antioxidant activity for both blackberry and grape; however, only the extracts obtained via Sox showed an inhibitory effect against *Escherichia coli* (IAL 2064) and *Staphylococcus aureus* (ATCC 13565). As for cell viability, the non-polar extracts did not show cytotoxicity or antiproliferative effect in the different cell lines, which shows their relative toxicological safety. Considering the requirements of Agenda 2030 and the need for sustainable ways to produce food ingredients, we have concluded that grape and blackberry seeds can be used as sources of derived lipid antioxidants in different technological applications.

## Figures and Tables

**Table 1:** Total phenolic content and antioxidant activity of non-polar blackberry and grape seed extracts.

	**Non-Polar Blackberry** **Seed Extracts**			
	**Sox**	**BD**	**US**	***p*-Value**
TPC (mg GAE/100 mL)	7.37 ± 0.25	102.37 ± 4.38	7.31 ± 0.30	<0.001
DPPH (mg AAE/100 mL)	6.91 ± 0.10	11.50 ± 0.44	2.07 ± 0.13	<0.001
TRC (mg QE/100 mL)	ND	46.42 ± 1.64	ND	<0.001
Fe^2+^ chelating ability (mg EDTAE/100 mL)	261.12 ± 12.13	17.12 ± 0.43	77.57 ± 0.12	<0.001
Yield (%)	20.7	13.3	7.7	
	**Non-Polar Grape** **Seed Extracts**			
	**Sox**	**BD**	**US**	***p*-Value**
TPC (mg GAE/100 mL)	7.21 ± 0.18	28.61 ± 0.50	7.01 ± 0.29	<0.001
DPPH (mg AAE/100 mL)	6.18 ± 0.39	35.36 ± 0.99	3.95 ± 0.20	<0.001
TRC (mg QE/100 mL)	ND	19.36 ± 0.34	125.89 ± 1.60	<0.001
Fe^2+^ chelating ability (mg EDTAE/100 mL)	155.01 ± 1.20	18.62 ± 0.13	74.70 ± 0.60	<0.001
Yield (%)	31.9	20.0	12.6	

Note: Sox = Soxhlet, BD = Bligh–Dyer, US = ultrasound, GAE = gallic acid equivalent, AAE = ascorbic acid equivalent, QE = quercetin equivalent, EDTAE = ethylenediamine tetraacetic acid equivalent, ND = not detected, TPC = total phenolic content, TRC = total reducing capacity.

**Table 2:** Individual fatty acid compounds and phytosterols of blackberry and grape seed extracts.

	**Non-Polar Blackberry** **Seed Extracts (mg/mL of Extract)**			
	**Sox**	**BD**	**US**	***p*-Value**
Palmitic acid (16:0)	1.00 ± 0.27	1.05 ± 0.14	0.98 ± 0.05	0.8746
Linolenic acid (18:3)	0.11 ± 0.02	0.12 ± 0.03	0.12 ± 0.01	0.7702
Linoleic acid (18:2)	6.88 ± 2.34	7.89 ± 0.98	7.69 ± 0.87	0.7155
Oleic acid (18:1)	4.38 ± 1.69	6.42 ± 1.68	5.31 ± 0.57	0.2824
Stearic acid (18:0)	0.44 ± 0.05	0.61 ± 0.22	0.29 ± 0.02	0.0605
Arachidic acid (20:0)	0.25 ± 0.13	0.45 ± 0.06	0.42 ± 0.01	0.0524
Monopalmitin	0.23 ± 0.05 ^a^	0.24 ± 0.03 ^a^	0.13 ± 0.01 ^b^	0.0086 ^*^
Monostearin	0.21 ± 0.06 ^ab^	0.26 ± 0.01 ^a^	0.15 ± 0.01 ^b^	0.0255 ^*^
Campesterol	0.19 ± 0.01 ^b^	0.21 ± 0.00 ^a^	0.21 ± 0.01 ^a^	0.0370
Stigmasterol	0.27 ± 0.01 ^c^	0.36 ± 0.02 ^a^	0.32 ± 0.02 ^b^	0.0012
β-sitosterol	3.11 ± 0.05 ^b^	3.55 ± 0.06 ^a^	3.62 ± 0.05 ^a^	<0.0001
β-Amyrenol	0.21 ± 0.01 ^b^	0.22 ± 0.01 ^b^	0.26 ± 0.01 ^a^	0.0006
Methylene cycloartenol	0.45 ± 0.02 ^b^	0.42 ± 0.02 ^c^	0.57 ± 0.01 ^a^	<0.0001
	**Non-Polar Grape** **Seed Extracts (mg/mL of Extract)**			
	**Sox**	**BD**	**US**	***p*-Value**
Palmitic acid (16:0)	1.47 ± 0.16 ^b^	1.80 ± 0.06 ^a^	1.75 ± 0.09 ^a^	0.0212
Linolenic acid (18:3)	0.38 ± 0.04	0.33 ± 0.01	0.38 ± 0.02	0.0872
Linoleic acid (18:2)	5.17 ± 1.20 ^b^	7.05 ± 0.24 ^ab^	8.19 ± 1.14 ^a^	0.0233
Oleic acid (18:1)	3.53 ± 0.62 ^b^	4.71 ± 0.30 ^ab^	5.43 ± 1.00 ^a^	0.0414
Stearic acid (18:0)	0.38 ± 0.04 ^c^	0.66 ± 0.02 ^a^	0.51 ± 0.08 ^b^	0.0023
Arachidic acid (20:0)	0.24 ± 0.02 ^a^	0.16 ± 0.01 ^b^	0.16 ± 0.02 ^b^	0.0009
Monopalmitin	0.09 ± 0.02	0.12 ± 0.01	0.10 ± 0.01	0.1725
Monostearin	0.03 ± 0.01 ^a^	0.05 ± 0.00 ^a^	0.03 ± 0.01 ^a^	0.0024
Campesterol	0.25 ± 0.01 ^b^	0.28 ± 0.02 ^b^	0.58 ± 0.09 ^a^	0.0004
Stigmasterol	0.24 ± 0.01 ^b^	0.29 ± 0.01 ^a^	0.22 ± 0.01 ^b^	0.0002
β-Sitosterol	1.3 ± 0.02 ^b^	1.56 ± 0.01 ^a^	1.22 ± 0.03 ^c^	<0.0001

Note: *, different upperscript letters in the same line represent statistically different results at *p* < 0.05.

## Data Availability

Not available.

## Sample Availability

Samples of the extracts are available from the authors.
